# *Bartonella quintana* endocarditis in a child from Northern Manitoba, Canada

**DOI:** 10.1371/journal.pntd.0010399

**Published:** 2022-05-26

**Authors:** Carl Boodman, William MacDougall, Michael Hawkes, Gregory Tyrrell, Sergio Fanella

**Affiliations:** 1 University of Manitoba, Winnipeg, Manitoba, Canada; 2 University of Alberta, Edmonton, Alberta, Canada; University of Pretoria, SOUTH AFRICA

## Abstract

We describe a case of *Bartonella quintana* endocarditis in an 11-year-old child from Northern Manitoba, Canada. This case demonstrates the neglected endemicity of *B*. *quintana* in Northern Canada and highlights the need for improved case finding and elucidation of specific risk factors for *B*. *quintana* infection in the Canadian North. Considering *B*. *quintana*’s predominant transmission via body lice ectoparasitosis, we hypothesize that *B*. *quintana*’s endemicity in Northern Canada is linked to inadequate access to suitable housing and running water among remote communities in the Canadian North.

## Case presentation

An 11-year-old male from a remote community in Northeastern, Manitoba presented to the cardiology clinic in Winnipeg, Manitoba with a 4-week history of fatigue, anorexia, and subjective weight loss. His medical history was notable for a bicuspid aortic valve and rheumatic fever diagnosed in 2019, treated with penicillin G prophylaxis every 28 days. He denied any recent fever, rash, upper respiratory tract symptoms, or joint swelling. He had a history of hunting grouse, but reported no other animal exposures. He lived in an Oji-Cree community where half of the households do not have running water and plumbing. There was no reported history of arthropod infestation.

On physical exam, the patient was in no acute distress. He was afebrile, and his vital signs were within normal limits. Cardiac auscultation revealed a grade III/VI diastolic murmur. Lung auscultation revealed basilar crackles bilaterally. Other notable exam findings included hepatomegaly and digital clubbing. Echocardiogram demonstrated new severe aortic insufficiency with a thickened aortic valve and an ejection fraction of 46%.

The patient was admitted to Winnipeg’s Health Sciences Centre (HSC) and treated as a relapse of rheumatic fever with penicillin V and methylprednisolone. This therapy was stopped when the patient’s antistreptolysin O titer (ASOT) was only 200 IU/mL. Computed tomography of the chest, abdomen, and pelvis demonstrated septic emboli to the spleen and left kidney. Magnetic resonance imaging of the brain was normal.

Blood cultures drawn on admission yielded no growth. Serology for HIV, *Brucella* species, and *Coxiella burnetii* were negative. Upon discovery of septic emboli, the patient was diagnosed with culture negative infective endocarditis and was started on ceftriaxone and vancomycin, 4 days after admission.

Ten days after admission, the patient developed worsening heart failure and was urgently transferred to the Stollery Children’s Hospital in Edmonton, Alberta. He underwent a Ross procedure the following day, and his aortic valve was successfully replaced. His antibiotics were changed to piperacillin–tazobactam and gentamicin. Pathology of the explanted aortic valve demonstrated vegetations over 80% of the tissue. The specimen demonstrated areas of fibromyxoid tissue, calcification, and patchy lymphocytic infiltrate. Gram stain of the tissue showed polymorphonuclear cells and no bacteria. *Bartonella quintana* was subsequently identified by 16S rRNA sequencing of a portion of the explanted aortic valve followed by DNA sequencing of the resulting amplicon. BLAST analysis of the DNA sequence demonstrated 100% homology to *B*. *quintana* (see S1 Appendix). Results for *B*. *quintana* serology, using a semiquantitative indirect immunofluorescent antibody assay (Focus Diagnostics), were subsequently reported as positive (IgG titer 1:1024) [1].

Seven days after surgery, the patient was transferred back to Winnipeg’s HSC where his antibiotics were changed to gentamicin (1 mg/kg/dose IV q8h) and doxycycline (100 mg PO BID). He received 4 weeks of gentamicin and 6 weeks of doxycycline. At follow-up 3 weeks and 2 months following discharge, the patient had no signs of heart failure or ongoing infection.

### Ethics statement

This case report was approved by the University of Manitoba (Bannatyne Campus) Research Ethics Board (HS25337:H2022:030). Formal written consent was obtained from the patient’s parent.

## Discussion

*B*. *quintana* is a fastidious, gram-negative, intraerythrocytic bacillus [2]. Due to its niche within erythrocytes and its slow replication time, *Bartonella* species evade identification by routine blood culture methods [2,3]. *B*. *quintana* is predominantly transmitted by body lice feces, entering the bloodstream through microabrasions of the skin [2]. While originally identified among soldiers in the trenches of World War I, *B*. *quintana* has emerged among urban populations experiencing homelessness, leading to the designation of “urban trench fever” [4,5]. *B*. *quintana* is a major cause of culture-negative endocarditis and is associated with chronic bacteremia that may last many months [6].

*B*. *quintana* has recently been reported in rural Northern Canada. The first published rural Canadian case of *B*. *quintana* endocarditis was acquired in Northern Manitoba in 2014 in a remote Cree community, 500 kilometers from the community where this pediatric patient lived [7]. In 2020, 2 additional cases of *B*. *quintana* endocarditis were diagnosed in individuals from a different Oji-Cree community, 75 kilometers from the one presented here [8]. In 2021, a fourth case of *B*. *quintana* endocarditis was described in a patient from rural Northern Alberta [9]. Our case represents the fifth published case of *B*. *quintana* endocarditis in rural Northern Canada, the first pediatric case of *B*. *quintana* in Canada and the first pediatric case of *B*. *quintana* endocarditis acquired in a high-income country [10].

In 2019, a case of urban *B*. *quintana* endocarditis was described in a man experiencing homelessness in a Canadian city [11]. This case, along with a review of 12 other North American cases of *B*. *quintana* endocarditis, occurred outside the hypothesized “Europe–African gradient” [11,12]. This concept describes a latitudinal gradient between areas of high *B*. *quintana* prevalence in Africa and low prevalence in Northern Europe [11,12]. The increasing number of *B*. *quintana* endocarditis cases in Northern Canada defy the “Europe–African gradient” and suggest that this gradient may be an economic one, rather a latitudinal one: Northern populations experiencing poverty may demonstrate similar rates of *B*. *quintana* to Southern populations.

Pediculosis was not described in any of the rural Canadian cases of *B*. *quintana* endocarditis. While *B*. *quintana* has been detected in alternate vectors such as cat fleas, most studies suggest that *B*. *quintana* is a specialist adapted to the louse vector and human host. [2,13–15] A nonhuman reservoir and nonlouse vectors remain possible, although undescribed in the Canadian context.

Due to its predominant link with body lice ectoparasitosis, *B*. *quintana* transmission occurs where there is inadequate access to suitable housing and running water, whether that be in historic wartime Europe, urban homeless encampments, or disadvantaged communities of Northern Canada [4,5,7–9]. The patient described here was housed and had not spent significant time in an urban center. As many indigenous communities in Canada are plagued by a housing and running water crisis, we suggest that living in a remote community in Northern Canada is a risk factor for *B*. *quintana* [16]. This case highlights the need for active *B*. *quintana* case finding and elucidation of specific risk factors for *B*. *quintana* acquisition in the Canadian North. We hypothesize that *B*. *quintana*’s endemicity in rural Northern Canada is linked to inadequate access to running water and neglected pediculosis in certain remote communities.

**Key Learning Points**

- *Bartonella quintana*, the bacterium causing trench fever, is predominantly transmitted via body lice, although other modes of transmission in rural Northern Canada have not been fully explored.
- *B*. *quintana* evades identification in routine blood cultures, and, thus, diagnosis of *B*. *quintana* requires serology and/or molecular testing and communication with the microbiology laboratory.
- *B*. *quintana* causes culture-negative endocarditis that may be fatal despite mild symptomatology during preceding prolonged bacteremia.
- While *B*. *quintana* is infamous for causing disease among soldiers in the First World War, as well as refugees in underserved camps and urban populations experiencing homelessness, this rural pediatric case highlights the neglected endemicity of trench fever in rural Northern communities in Canada.

## Supporting information

S1 Appendix DNA sequence of 16S rRNA gene and its associated database search results and DNA alignment first hits. (DOCX)
