# Supplementary material for: Bartonella quintana endocarditis in a child from Northern Manitoba, Canada
Source: PLoS Negl Trop Dis. 2022 May 26;16(5):e0010399. doi: 10.1371/journal.pntd.0010399 (PMC9135198; doi:10.1371/journal.pntd.0010399)
Supplement: S1 Appendix — (DOCX) [file pntd.0010399.s001.docx]

**Appendix 1. DNA sequence of 16S rRNA gene and its associated database search results**

Below is the DNA sequence of a portion of the 16S rRNA gene of the isolate identified as *Bartonella quintana*. One strand of the 16S rRNA gene was amplified from the tissue by PCR and sequenced.

GCAAGTCGAGCGCACTCTTTTAGAGTGAGCGGCAAACGGGTGAGTAACGCGTGGGAATCTACCCATCTCTACGGAATAACACAGAGAAATTTGTGCTAATACCGTATACGTCCCTCTGGGAGAAAGATTTATCGGAGGTGGATGAGCCCGCGTTGGATTAGCTAGTTGGTGAGGTAAGGGCTCACCAAGGCGACGATCCATAGCTGGTCTGAGAGGATGATCAGCCACACTGGGACTGAGACACGGCCCAGACTCCTACGGGAGGCAGCAGTGGGGAATATTGGACAATGGGGGCAACCCTGATCCAGCCATGCCGCGTGAGTGATGAAGGCCCTAGGGTTGTAAAGCTCTTTCACCGGTGAAGATAATGACGTTAACCGGAGAAGAAGCCCCGGCTAACTTCGTGCCAGCAGCCGCGGTAATACGAAGGGGGCTAGCGTTGTTCGGATTTACTGGGCGTAAAGCGCATGTAGGCGGATATTTAAGTCAGAGGTGAAATCCCAGGGCTCAACCCTGGAACTGCCTTTGATACTGGATGTCTCGAGTGTGGAAGAGGTGAGTGGAATTCCGAGTGTAGAGGTAAAATTCGTAGATATTCGGAGGAACACCAGTGGCGAAGGCGGCTCACTGGTCCATTACTGACGCTGAGGTGCGAAAGCGTGGGGAGCAAACAGGATTAGATACCCTGG

Two databases were searched:

1. RDP: ribosomal database project (RDP)
2. NCBI

Both provided the same results.

The search results when the unknown 16S sequence was searched using the ribosomal database project (RDP) search tool. Website: <https://www.rdp.cme.smu.edu>

Best hit was for *Bartonella quintana* at 1.000 for most *B. quintana* isolates.

BLAST search using the NCBI BLAST tool. The top hits for the input sequence of 689 nucleotides were *Bartonella quintana* 16S rDNA genes.

DNA alignment of the first hit (species) from the previous BLAST output list. For the 689 nucleotides searched, the match was 100%.
